# Editorial: Plant-microbe interactions in forest ecosystems, volume II

**DOI:** 10.3389/fpls.2024.1414383

**Published:** 2024-04-25

**Authors:** Julio Javier Diez Casero, Ana Paula Moreira Rovedder, Luciano Kayser Vargas

**Affiliations:** ^1^ Sustainable Forest Management Research Institute (IuFOR), University of Valladolid and INIA, Palencia, Spain; ^2^ Department of Forest Sciences, Federal University of Santa Maria, Santa Maria, Brazil; ^3^ Laboratório de Química Agrícola, Departamento de Diagnóstico e Pesquisa Agropecuária (DDPA), Secretaria Estadual da Agricultura, Pecuária e Desenvolvimento Rural, Porto Alegre, Brazil

**Keywords:** forest, mycorrhizal fungi (MF), microbial diversity, microbial ecology, phytopathogenic fungi

In an era in which human activities are increasingly affecting the planet and contributing to climate change, forest environments, whether natural or planted, acquire strategic importance. From an economic point of view, forests yield traditional products, such as wood and cellulose, and offer a series of potential non-wood items. From an environmental point of view, forests act as a refuge for native flora and fauna, helping to preserve biodiversity. Furthermore, trees promote carbon sequestration by incorporating carbon dioxide from the atmosphere into plant biomass and soil. This process mitigates greenhouse gas emissions and enhances soil and water quality. It is estimated that forests and their soils accumulate more than 40% of terrestrial carbon (Wei et al., 2014), exceeding the amount present in the atmosphere (Sarkodie et al., 2023).

Compared to agricultural soils, forest soils are characterized by their higher carbon accumulation (Paul et al., 2023) and intense microbial activity (da Silva et al., 2012). Microorganisms in forest soils are the main drivers of biogeochemical cycles, promoting the cycling of nutrients and organic matter. They also form intricate symbiotic relationships with forest species, such as the mutualistic associations between rhizobia and leguminous trees or mycorrhizal fungi and conifers. On the other hand, phytopathogenic microorganisms can drastically reduce forest production. Therefore, understanding the relationships between forest species and soil microorganisms is essential for better management of these ecosystems. By understanding how different forest management and species impact microbial diversity and activity, as well as identifying and exploring the positive and negative interactions between microorganisms and forest species, we can use these microorganisms as technological tools that can make forestry production more efficient and ecologically friendly, from seedling production to the end of the forestry cycle.

As mentioned, negative interactions between plants and microorganisms may occur. These interactions include the competition for nutrients and, most notably, the incidence of plant diseases. In this context, Dobbs et al. studied the diversity of fusarioid fungi associated with damping-off and root diseases of conifer seedlings throughout the contiguous USA. By evaluating two gene sequences, tef1a and rpb2, the authors surveyed the nurseries to identify patterns of geographic distribution and host range across fusarioid fungi, as well as to determine if fungi haplotypes were widespread or isolated within a region. The results indicated that some fusarioid fungi haplotypes were associated with host and geographic location specificity. On the other hand, generalist fungi were found on several hosts in different geographical regions.

The relationship between phytopathogenic fungi and trees was also the subject of research conducted by Ahmad and Diez. The authors investigated the genetic diversity of *Cryphonectria parasitica* and *Fusarium circinatum*, two invasive disease-causing fungi introduced to Europe from North America at different times. *C. parasitica* causes chestnut blight disease and reached Spain about nine decades ago, while *F. circinatum* arrived about two decades ago and causes pine pitch canker. The authors hypothesized that the initial fungus exhibits greater genetic diversity owing to its extended duration for genetic alterations. Molecular genetic markers were employed to validate this hypothesis, which was substantiated by the higher genetic diversity observed in *C. parasitica*. This investigation contributes to elucidating the invasion dynamics of both fungi in northern Spain and offers valuable insights to bolster biological control strategies against these pathogens.

Mycorrhizal fungi are ubiquitous root symbionts that play a key role in terrestrial ecosystems by enhancing nutrient uptake by plants. Ectomycorrhizae (ECM) predominate in temperate forests, while arbuscular mycorrhizae (AM) are most common in the tropics, and both types may occur in comparable abundance in subtropical regions (Toju et al., 2014). Tanunchai et al. analyzed how the different tree mycorrhizal species regulate the structure of the microbial communities of leaves and needles and influence the decomposition rate of litter in temperate forests. The authors employed high-throughput sequencing techniques to analyze the microbial community associated with nine temperate tree species, encompassing broadleaved arbuscular mycorrhizal, broadleaved ectomycorrhizal, and coniferous ectomycorrhizal tree species, throughout the process of leaf litter decomposition. Their findings indicate that the mycorrhizal types of trees exert a significant influence on leaf litter quality, thereby impacting microbial richness and community composition, ultimately affecting the rate of leaf litter decomposition.

Ectomycorrhizae play a fundamental role in the growth of *Cedrus deodara*. Wen et al. investigated the ECM colonization status of four urban *C. deodara* forests in eastern China. They identified nineteen ECM fungal species, of which thirteen species were found in mature forests, and nine species were identified in bioassay experiments, with only three species co-occurring. Species abundance was influenced by soil pH and P availability. The abundance of ECM fungi affected seedling growth and promoted significant root growth, highlighting the importance of mycorrhizal colonization for conserving this important tree species.

Understanding the interactions between plants and soil microorganisms is crucial for conserving the biodiversity and the functioning of forest ecosystems. In their review article, Maitra et al. addressed the influence of root exudates on microbial diversity, with particular attention to global climate change. Variability in the exudation pattern of tree roots can affect microbial abundance and diversity. In turn, climatic conditions influence these exudation patterns, creating ecological niches for diverse microbial communities. The review highlights how forest plants can adapt to challenging conditions using their root traits to establish positive interactions with microbes.

Soil microorganisms may accelerate seed germination and even break the dormancy of hard seeds. Wu et al. screened out and isolated more than 100 different culturable microorganisms that could rapidly erode the pericarp of *Tilia miqueliana*, a Chinese tree species. The authors also evaluated the microbial diversity in the soil samples and assembled mixed artificial cultures with microorganisms that could promote cracking in the pericarp without damaging the embryos inside the fruits.

Nowadays, the preservation of biodiversity is of great relevance and microbiota needs to be part of the debate on the management of terrestrial and aquatic ecosystems, given the ecosystem services it provides. We expect that this compilation of articles will encourage further discussions and inspire newer research on this subject.

## Author contributions

JC: Writing – original draft, Writing – review & editing. AR: Writing – original draft, Writing – review & editing. LV: Writing – original draft, Writing – review & editing.
